# When Chronic Risk Meets Acute Strain: Sudden Cardiac Death and Cardiovascular Vulnerability in Firefighters

**DOI:** 10.1177/11795468261479323

**Published:** 2026-08-11

**Authors:** Ibrahim Mortada, Ebrahim Adel Elkolaly, Aaron W. Lee, Dalton Buckingham, Diann Gaalema, Mostafa Shalaby, Michael C. Boyars, Thomas A. Blackwell, Hani Jneid

**Affiliations:** 1 Department of Cardiovascular Medicine, 12338 University of Texas Medical Branch, Galveston, TX, USA; 2 School of Medicine, Badya University, Cairo, Egypt; 3 John Sealy School of Medicine, 12338 University of Texas Medical Branch, Galveston, TX, USA; 4 Department of Internal Medicine, 12338 University of Texas Medical Branch, Galveston, TX, USA

**Keywords:** cardiovascular disease, firefighters, cardiometabolic risk, sudden cardiac death, occupational exposure, autonomic nervous system, coronary artery calcium, heart rate variability, post-traumatic stress disorder

## Abstract

Cardiovascular disease remains the leading cause of line-of-duty mortality among firefighters. Sudden cardiac death occurs disproportionately during emergency operations, particularly fire suppression, suggesting an interaction between occupational stressors and underlying cardiovascular vulnerability. This narrative review integrates epidemiologic, mechanistic, and clinical data from observational studies, systematic reviews, and contemporary cardiovascular guidelines. A structured literature search of PubMed and Google Scholar identified studies addressing cardiovascular disease, sudden cardiac death, occupational stress, and autonomic regulation in firefighters. Autopsy and case–control data indicate that most duty-related sudden cardiac deaths occur in the presence of underlying coronary atherosclerosis. Firefighters exhibit a high prevalence of cardiovascular risk factors, including hypertension (10%–44%), obesity (14%–60%), dyslipidemia (20%–56%), and metabolic syndrome (∼22%); these rates exceed those reported in age-matched general-population samples. Emergency operations are associated with marked sympathetic activation, hemodynamic stress, and circadian perturbation, which may promote plaque destabilization, myocardial ischemia, and ventricular arrhythmia. Additionally, occupational stress and sleep disturbance are linked to autonomic imbalance, potentially increasing arrhythmic susceptibility. Sudden cardiac death in firefighters reflects the interaction between baseline cardiometabolic risk and intermittent high-intensity physiologic stress rather than a distinct occupational disease entity. Early risk identification, guideline-directed therapy, and recognition of occupational stressors are central to cardiovascular risk reduction in this population.

## 1. Introduction

National surveillance analyses from the United States Fire Administration indicate that approximately 100 firefighters die annually in the United States while on duty.^1^ Cardiovascular disease, particularly coronary heart disease (CHD), remains the leading cause of this line-of-duty mortality. Kales et al. demonstrated that cardiovascular events account for approximately 45% of on-duty firefighter fatalities in the United States, exceeding deaths from traumatic causes such as burns and structural collapse.^2,3^ These observations raise important questions regarding whether current occupational fitness standards and periodic medical screening provide adequate protection against cardiovascular events.

Firefighting, as an occupation, serves as a cardiovascular stress test. The job is characterized by prolonged intervals of relative inactivity punctuated by abrupt transitions to near-maximal effort exertion under extreme environmental conditions. Fire suppression requires sustained high-intensity activity while wearing insulated personal protective equipment that may exceed 25–30 kg. This exertion is often performed in high-temperature environments that impose substantial hemodynamic and thermal stress.^3-5^ These demands are further compounded by psychological stress and unpredictable alarm response, resulting in repeated surges in heart rate, blood pressure, and sympathetic activation.^3^ Moreover, the risk of CHD death is markedly elevated during emergency duties, particularly fire suppression, with relative risk estimates ranging from 10- to 100-fold compared with non-emergency duties.^2,4^

However, acute occupational stressors alone do not fully explain the observed patterns of sudden cardiac death. Epidemiological data indicate that many affected firefighters have underlying cardiometabolic risk factors or previously unrecognized structural cardiovascular disease.^4,6,7^ These combinations of findings support a model in which occupational stress functions as a trigger superimposed upon pre-existing cardiovascular vulnerability, a framework previously articulated by Smith et al. in their review of sudden cardiac death in the U.S. fire service.^8^ In parallel, repeated exposure to traumatic events and chronic occupational stress has been associated with autonomic imbalance, sleep disturbance, and psychological morbidity, including post-traumatic stress disorder,^9-14^ factors which may lower the threshold for ischemic and arrhythmic events during periods of acute physiological stress.

Collectively, available evidence supports a conceptual framework in which sudden cardiac death in firefighters arises from the interaction between chronic cardiometabolic risk and intermittent high-intensity occupational stress. Baseline risk provides the structural substrate, while acute physiologic and psychological stressors contribute to autonomic dysregulation and precipitate cardiovascular events.

In this narrative review, we examine contemporary evidence regarding cardiovascular health in firefighters, with emphasis on cardiovascular mortality, cardiometabolic risk burden, psychological stress and autonomic dysregulation, and the pathophysiological mechanisms underlying sudden cardiac death. We further discuss implications for cardiovascular screening and preventive strategies in this occupational population.

### 1.1. Literature Search and Scope

Because the present work is a narrative rather than a systematic review, no protocol was registered. We interrogated PubMed and Google Scholar from database inception through 2025, combining terms such as “firefighter, cardiovascular disease, coronary heart disease, sudden cardiac death, line-of-duty death, cardiometabolic risk, metabolic syndrome, occupational stress, heart rate variability, autonomic function, and coronary artery calcium”. Preference was given to peer-reviewed observational studies, systematic reviews and meta-analyses, autopsy and case–control reports, and current cardiovascular guidelines, and the reference lists of retrieved articles were examined for further relevant work. Reports lacking primary or synthesized cardiovascular data, and publications not available in English, were not considered.

Although it is well recognized that sudden cardiac death in firefighters reflects an interplay between pre-existing cardiovascular disease and acute occupational stress, the present review seeks to build on earlier accounts in several ways. It draws contemporary cardiometabolic prevalence data together with mechanistic evidence on autonomic dysregulation, disturbed sleep, and psychological trauma within a single explanatory framework; it incorporates recently reported firefighter-specific coronary artery calcium data; and it uses this synthesis to outline an individualized approach to cardiovascular screening and fitness-for-duty assessment in place of a uniform testing strategy.

## 2. Overall Cardiovascular Mortality Burden

Kales et al. demonstrated that cardiovascular disease accounts for approximately 45% of line-of-duty deaths among firefighters, making it the leading cause of duty-related mortality in this population.^2,4^ In comparison, cardiac causes account for a smaller proportion of occupational deaths among other emergency service personnel, including approximately 22% among police officers and 11% among emergency medical services personnel.^1,15^

The task-specific distribution of these events provides important insight into their underlying mechanisms. Duty-related cardiac deaths occur predominantly during emergency operations rather than routine station activities. Activation for fire suppression is associated with an approximately 10- to 100-fold higher risk of coronary death compared with non-emergency duties, with similar elevations observed during alarm response and strenuous physical training.^2,4^ Among younger firefighters, aged ≤45 years, fire suppression duties have been associated with a more than 20-fold increased risk of sudden cardiac death compared with non-emergency duties.^16^ The incidence of duty-related coronary death increases with age, with higher rates observed among older firefighters. The circadian distribution of events parallels patterns of emergency dispatch activity, supporting an association between periods of occupational demand and increased cardiovascular risk.^2^

Autopsy-based analyses further characterize the underlying structural disease burden in affected individuals. Approximately 80% of firefighters who experienced duty-related cardiac death have evidence of coronary heart disease at autopsy, frequently accompanied by cardiomegaly or left ventricular hypertrophy.^4^ Case–control studies similarly demonstrate a high prevalence of coronary artery disease and major cardiovascular risk factors such as hypertension, diabetes, and smoking.^6,7^

Collectively, these findings indicate that firefighters represent an occupational group with substantial duty-related cardiovascular mortality, characterized by CHD as the leading cause, clustering during high-exertion tasks, and frequent coexistence of cardiometabolic risk factors. These findings provide an epidemiologic foundation for understanding the structural and physiologic mechanisms underlying acute cardiac events in this population.

## 3. Cardiometabolic Risk and Structural Substrate

### 3.1. Cardiometabolic Risk Burden

Firefighters exhibit a high prevalence of traditional cardiovascular risk factors across diverse geographic regions and service settings, including obesity (14%–60%), hypertension (10%–44%), dyslipidemia (20%–56%), and tobacco use (11%–39%).^17-19^ Current tobacco use among U.S. adults is estimated at approximately 11%–14%, making the upper range of firefighter tobacco prevalence substantially higher than the general population. Obesity is common and is associated with elevated blood pressure and adverse lipid profiles.^20-23^ Dyslipidemia and tobacco use also remain prevalent,^20,24-26^ contributing to overall atherosclerotic risk. Hypertension is particularly common and closely associated with cardiovascular morbidity.^20,21,27^ Given its established role in the progression of atherosclerotic disease and sudden cardiac death,^2^ suboptimal blood pressure control represents an important modifiable contributor to risk. The prevalence ranges for these risk factors across firefighter cohorts are summarized in Table 1.

**Table 1.** Prevalence of Cardiovascular Risk Factors in Firefighters

Risk factor	Reported prevalence	Key references
Obesity	14–60%	Ras et al.^16^; Soteriades et al.^6^
Hypertension	10–44%	McMorrow et al.^26^; Choi et al.^20^
Dyslipidemia	20–56%	Ras et al.^16^; Drew-Nord et al.^17^
Tobacco use	11–39%	Jitnarin et al.^28^
Metabolic syndrome	∼22%	Beckett et al.^18^

Prevalence ranges reflect findings from multiple firefighter cohorts reported in the cited studies.

Cardiometabolic risk among firefighters is not static. Longitudinal data demonstrate progressive increases in body weight and overall risk factor burden over years of service; while weight gain with aging is not exclusive to this occupation, shift-work schedules, sleep disruption, and prolonged periods of standby inactivity may compound this trajectory beyond what is attributable to aging alone.^27^ Cross-sectional analyses further show increasing risk with age, with peak burden observed in mid-career firefighters (approximately 45–50 years).

Clustering of cardiometabolic abnormalities is also common. A meta-analysis including more than 30,000 firefighters reported a pooled prevalence of metabolic syndrome of approximately 22%.^19^, Hypertension and central adiposity appear to be predominant components. Although this estimate is numerically lower than general-population estimates of approximately 30%–35% among adults of similar age,^27^ it should not be interpreted as reassuring. Firefighters represent an occupationally selected workforce expected to meet physical performance standards and, in many jurisdictions, undergo periodic medical or fitness evaluations. Therefore, a metabolic syndrome prevalence approaching one in five firefighters is clinically concerning and suggests that operational fitness does not reliably exclude underlying cardiometabolic disease. Higher mean body mass index is associated with increased metabolic syndrome prevalence,^19^ supporting a central role of excess adiposity in risk clustering. The coexistence of hypertension, insulin resistance, and dyslipidemia has been linked to increased atherosclerotic burden and heightened susceptibility to coronary events. Notably, occupational fitness requirements do not preclude the presence of cardiometabolic disease, and ability to pass physical performance tests may not reflect underlying cardiovascular risk.^29,30^

### 3.2. Structural Substrate for Acute Events

The cardiometabolic profile observed in firefighters suggests that many individuals who experience duty-related cardiac events have underlying structural cardiovascular disease. Autopsy and case–control analyses have frequently demonstrated coronary atherosclerosis or left ventricular hypertrophy.^4,6,7^ These observations indicate that sudden cardiac death most often occurs in the setting of established pathology rather than structurally normal myocardium.

From a pathophysiologic perspective, the coexistence of obesity, hypertension, dyslipidemia, and metabolic syndrome promotes endothelial dysfunction and the development of atherosclerosis. The underlying substrate may remain clinically unrecognized but can predispose to myocardial ischemia or malignant ventricular arrhythmias when exposed to acute physiologic stress. Accordingly, cardiometabolic abnormalities represent a structural substrate underlying cardiovascular events in this population.

## 4. Psychophysiological Stress and Autonomic Dysregulation

### 4.1. Occupational Stress and Sympathetic Activation

Firefighting involves recurrent exposure to high-intensity and unpredictable occupational stressors, including emergency response activities and traumatic operational environments. In addition to acute demands, organizational structure and work conditions contribute to chronic occupational stress. Elevated stress levels have been associated with alterations in autonomic regulation, particularly reductions in parasympathetic modulation.^9^

Both physical exertion and psychological stress are associated with increased sympathetic activity and reduced vagal tone.^31-35^ These changes are reflected by decreased heart rate variability and attenuation of high-frequency components corresponding to parasympathetic activity. In individuals with hypertension, autonomic imbalance appears more pronounced, consistent with impaired buffering capacity.^32^ Given the high prevalence of hypertension among firefighters,^27,36^ recurrent sympathetic activation may have amplified physiologic effects in this population.

### 4.2. Heart Rate Variability, Sleep Disturbance, and Cardiovascular Risk

Heart rate variability (HRV) is a noninvasive index of autonomic regulation, and reduced HRV has been associated with increased mortality in population-based studies.^37^ Although HRV is not disease-specific, diminished variability reflects impaired autonomic modulation and may be associated with increased susceptibility to ventricular arrhythmia and myocardial ischemia.^2,37^ Among firefighters, occupational stress has been associated with reductions in HRV indices, independent of traditional cardiovascular risk factors.^9^

Occupational sleep disruption represents an additional, sustained contributor to autonomic imbalance. Sleep disturbance, which is common among firefighters, contributes to sympathetic predominance and impaired parasympathetic recovery. Shift work and circadian disruption further affect autonomic regulation.^37^ Circadian variation in sympathetic activity, particularly the early morning surge, has been implicated in the timing of cardiovascular events.^31,38-40^ In individuals with elevated cardiometabolic risk, these factors may contribute to persistent autonomic imbalance and increased vulnerability during periods of exertion.

### 4.3. Psychological Trauma and Cumulative Exposure

Post-traumatic stress disorder (PTSD), depression, and anxiety are frequently reported among first responders.^10-12,14^ These conditions reflect both acute traumatic exposures and cumulative occupational stress.^37^ Chronic stress and hyperarousal are associated with sustained sympathetic activation and reduced parasympathetic tone. Although interventions such as cognitive behavioral therapy can reduce symptom burden,^13^ physiologic stress responses may persist, highlighting a distinction between psychological improvement and physiologic restoration of autonomic regulation.

### 4.4. Integrative Implication

Firefighters are exposed to repeated sympathetic activation related to acute exertion, chronic stress, sleep disruption, and psychological trauma. These factors are associated with substantial alterations in autonomic regulation that may increase susceptibility to arrhythmic events and hemodynamic instability during periods of high physiologic demand.

## 5. Pathophysiological Mechanisms of Sudden Cardiac Death in Firefighters

### 5.1. Coronary Atherosclerosis as Structural Substrate

**Figure 1.**
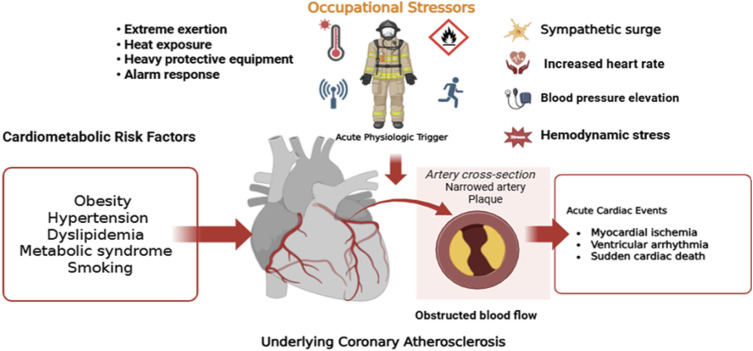
Conceptual framework illustrating the interaction between chronic cardiometabolic risk factors and occupational stressors to precipitate acute cardiac events in firefighters

Epidemiologic and autopsy data indicate that most duty-related sudden cardiac deaths in firefighters occur in the setting of underlying CHD rather than primary electrical disorders in structurally normal myocardium.^2,3,6,7^ Structural findings, particularly coronary atherosclerosis and, in some cases, left ventricular hypertrophy are documented in the majority of affected individuals and are discussed in detail in Sections II and III. The mechanistic focus here is on how occupational stressors interact with this substrate to precipitate acute events. This proposed interplay between the chronic cardiometabolic substrate and acute occupational triggers is depicted in Figure 1.

### 5.2. Hemodynamic Stress and Plaque Destabilization

Plaque disruption with superimposed thrombosis is a central mechanism underlying type 1 myocardial infarction.^41^ Sudden increases in heart rate and blood pressure during emergency response are associated with substantial hemodynamic stress. Concurrent catecholamine release increases myocardial oxygen demand and may contribute to transient coronary vasoconstriction. In firefighters with underlying coronary atherosclerosis, these physiologic changes may promote plaque instability and myocardial ischemia. The clustering of events during fire suppression, alarm response, and strenuous physical training^2-4,16^ is consistent with this mechanism. In addition to physical and hemodynamic demands, firefighters are exposed to combustion products including carbon monoxide and ultrafine particles, which may promote systemic inflammation, endothelial dysfunction, and impaired myocardial oxygen delivery, potentially compounding ischemic risk.^42,43^

### 5.3. Sympathetic Predominance and Arrhythmic Susceptibility

Acute exertion and psychological stress increase sympathetic activity and reduce vagal modulation.^31-35^ In individuals with limited autonomic reserve, these changes may compromise electrical stability. Reduced HRV, reflecting impaired parasympathetic regulation, has been associated with adverse cardiovascular outcomes.^37^ Among firefighters, occupational stress and sleep disturbance contribute to reductions in HRV,^9,28^ potentially increasing susceptibility to ventricular arrhythmia, particularly in the presence of myocardial ischemia or structural heart disease.

### 5.4. Ischemia–Arrhythmia Interaction

Myocardial ischemia and ventricular arrhythmia are closely interrelated processes.^2,37^ Acute ischemia increases electrical heterogeneity and alters myocardial refractory properties, promoting arrhythmogenesis.^31,37^ In firefighters with coronary atherosclerosis, the combination of increased myocardial oxygen demand, reduced perfusion, and potential plaque-related thrombosis may precipitate ventricular arrhythmias.^2,4,41^ This interaction may explain the abrupt clinical deterioration observed in many duty-related fatalities.^2^

### 5.5. Mechanistic Integration

These mechanisms align with established models of ischemic and arrhythmic death, in which underlying structural disease and acute physiological stress converge to precipitate clinical events. The principal occupational exposures considered above, with their physiologic correlates and possible cardiovascular sequelae, are collated in Table 2.

**Table 2.** Occupational Exposures in Firefighters, Associated Physiologic Responses, and Potential Cardiovascular Consequences

Occupational exposure	Physiologic response	Potential cardiovascular Consequence
Fire suppression operations	Extreme physical exertion, heat stress, dehydration	Increased myocardial oxygen demand and hemodynamic strain
Alarm response and emergency dispatch	Acute sympathetic activation and abrupt heart rate elevation	Plaque destabilization and ischemia
Heavy protective equipment	Increased cardiac workload and impaired heat dissipation	Hemodynamic stress
Shift work and sleep disruption	Circadian misalignment and sympathetic predominance	Autonomic imbalance and arrhythmic susceptibility
Psychological trauma and chronic stress	Sustained sympathetic activation and reduced parasympathetic tone	Increased risk of arrhythmia and ischemia
Combustion product inhalation (carbon monoxide, ultrafine particles)	Systemic inflammation, endothelial dysfunction, impaired oxygen delivery	Accelerated atherosclerosis, acute coronary syndrome risk

## 6. Screening and Preventive Strategies

### 6.1. Early Identification of Cardiometabolic Risk

Given that duty-related cardiac events in firefighters frequently occur in the setting of established structural cardiovascular disease,^2,4,6,7^ early identification and management of modifiable risk factors are central to risk reduction. Despite periodic medical evaluations in many jurisdictions, hypertension and cardiometabolic abnormalities remain prevalent and are often suboptimally controlled.^27,36^

Systematic blood pressure assessment, including consideration of out-of-office or ambulatory monitoring when appropriate, may improve detection of masked or stress-related hypertension. Considering the established association between elevated blood pressure and cardiovascular morbidity,^27^ adherence to contemporary hypertension management standards is particularly important in this population.

Smoking cessation represents an additional high-priority intervention. Tobacco use remains prevalent in some firefighter cohorts^44^ and contributes independently to atherosclerotic risk and sudden cardiac death. Brief cessation counseling and pharmacotherapy should be incorporated into routine occupational health evaluations.

Identification of dyslipidemia, impaired glucose metabolism, and central adiposity should prompt guideline-directed risk modification. The clustering of cardiometabolic abnormalities observed among firefighters^14^ supports an integrated risk assessment approach rather than management of isolated parameters.

### 6.2. Risk Stratification and Coronary Disease Management

For firefighters with established coronary artery disease or prior acute coronary syndromes, implementation of guideline-directed secondary prevention remains essential. High-intensity statin therapy, optimization of blood pressure control, lifestyle modification, and participation in structured cardiac rehabilitation represent core components of care.^41^ Given the substantial physical demands of firefighting, attainment of recommended secondary prevention targets may carry particular occupational relevance.

In selected individuals with elevated risk profiles, further cardiovascular evaluation such as assessment of functional capacity, testing for inducible ischemia, or measurement of lipoprotein(a) [Lp(a)] in those with a family history of premature coronary artery disease may assist in determining fitness for continued duty. Coronary artery calcium (CAC) scoring provides a noninvasive measure of subclinical atherosclerosis and has demonstrated prognostic value in asymptomatic populations. Among firefighters, CAC screening has been explored as a method to identify individuals with occult coronary disease not detected through traditional risk assessment. In a cohort of asymptomatic firefighters aged 40 years or older, CAC scores in a range associated with elevated cardiovascular risk were identified in approximately 39% of participants, with the majority of these individuals falling below conventional ASCVD pooled cohort equation thresholds that would have prompted preventive therapy, suggesting that traditional risk scoring may underestimate risk in some firefighters.^45,46^ These observations suggest that individualized risk stratification approaches may be warranted in selected individuals.

It warrants emphasis, however, that CAC scoring is not intended as a blanket test applied to every firefighter. Its yield is greatest in asymptomatic individuals, typically those aged 40 years or older, whose estimated ASCVD risk is borderline or intermediate, or who carry additional risk-enhancing features. Applied in this manner, CAC scoring informs rather than supplants the pooled-cohort ASCVD estimate; an absent calcium burden may justify deferring pharmacotherapy, whereas detectable calcification can reveal atherosclerosis that the standard equations underestimate in this group. Recent firefighter-specific studies lend further weight to this targeted use. In one cross-sectional cohort, advancing age, a higher circulating monocyte fraction, and raised alkaline phosphatase were each accompanied by greater coronary calcification, with alkaline phosphatase standing out as an independent correlate.^47^ A companion analysis from the same investigators noted that, among firefighters at or above 45 years of age, greater aerobic capacity tracked with a lower calcium burden, whereas no equivalent relationship was apparent in younger personnel.^48^ Read together, these findings favor an imaging-based approach to risk assessment and serve as a reminder that robust occupational fitness does not assure freedom from subclinical coronary disease. Several questions nevertheless remain open, among them the cost-effectiveness of such screening within the fire service, the cumulative radiation involved, the further testing and attendant risk prompted by positive or incidental findings, and the weight that calcium results should carry in return-to-duty and fitness-for-duty decisions. Until firefighter-specific outcome and implementation data are available, a selective and individualized strategy appears most appropriate.

### 6.3. Fitness and Body Composition

Physical performance is integral to operational safety; however, functional capability does not necessarily indicate absence of cardiometabolic disease.^29,30^ Maintenance of aerobic capacity and favorable body composition should therefore be considered components of cardiovascular risk reduction in addition to occupational performance requirements.

Structured wellness initiatives emphasizing sustained weight management, aerobic conditioning, and metabolic health may contribute to long-term risk reduction. These strategies should complement, rather than replace, formal cardiovascular risk assessment and evidence-based medical therapy when indicated.

### 6.4. Autonomic and Psychological Considerations

Occupational stress and sleep are associated with alterations in autonomic regulation among firefighters.^9,28^ Although routine clinical application of heart rate variability for risk prediction remains investigational, recognition of sustained occupational stress has practical clinical implications. Assessment for sleep disorders, depressive and anxiety symptoms, and PTSD^10-14^ may help identify individuals in whom persistent sympathetic activation and impaired autonomic recovery could contribute to overall cardiovascular risk.

Psychological interventions, including structured cognitive behavioral therapy, have been shown to reduce PTSD symptom severity.^13^ While direct data linking these interventions to cardiovascular outcomes is limited, reduction of chronic stress burden may be considered as part of a comprehensive risk reduction strategy.

### 6.5. Systems-Level Response to Acute Events

Despite preventive efforts, acute cardiovascular events will continue to occur. Automated external defibrillators (AEDs) should be readily accessible in fire station environments; although firefighters are trained in CPR and emergency response, rapid on-site defibrillation capability represents a critical system-level safeguard. Contemporary guidelines emphasize early symptom recognition, prompt electrocardiographic evaluation, and timely reperfusion when indicated.^41^ In firefighters, where events frequently occur during active duty, structured protocols facilitating rapid identification of symptoms, early ECG acquisition, and expedited transfer to appropriate care may improve outcomes.

For personnel with known coronary artery disease, adherence to guideline-directed therapy and appropriate cardiovascular evaluation prior to return to unrestricted operational duties remain important considerations.^41^

### 6.6. Integrated Preventive Perspective

Effective risk reduction in firefighters requires a coordinated approach emphasizing early identification of cardiometabolic abnormalities, implementation of guideline-directed medical therapy, maintenance of physical fitness, and recognition of occupational factors such as stress and sleep disruption. Integration of occupational health services, primary care, and cardiovascular specialists may facilitate structured risk assessment and longitudinal management in this population.

## 7. Limitations

This review has several limitations. As a narrative rather than systematic synthesis, it was not carried out under a registered protocol with dual independent screening, so selection and reporting bias cannot be excluded. Much of the supporting evidence is cross-sectional or derived from autopsy and case–control analyses, which constrains causal inference regarding the links between cardiometabolic burden, autonomic dysregulation, and acute cardiac events. The prevalence estimates cited are drawn from cohorts that differ appreciably in geography, age, and sex distribution, as well as in how risk factors were ascertained, and the predominance of data from male firefighters in the United States limits generalizability to female, volunteer, and non-U.S. personnel. Firefighter-specific studies linking coronary artery calcium, heart rate variability, or psychological treatment to adjudicated cardiovascular events likewise remain scarce, so several of the screening and preventive suggestions offered here are necessarily extrapolated from general-population data. Finally, the occupational and guideline landscape continues to evolve, and certain estimates and recommendations will require revision as further evidence accumulates.

## 8. Future Directions

Although the association between firefighting duties and sudden cardiac death has been consistently demonstrated,^2,4^ important gaps remain. Prospective data examining longitudinal cardiometabolic trajectories in relation to adjudicated cardiovascular events are limited, with most existing evidence derived from cross-sectional assessments or retrospective analyses. Long-term cohort studies incorporating serial risk factor assessment and standardized outcome definitions may provide greater insight into temporal risk evolution.

The role of autonomic dysfunction warrants further investigation. While reductions in heart rate variability have been described in firefighters exposed to occupational stress,^9^ the extent to which these measures independently predict ischemic or arrhythmic events remains uncertain. Prospective studies evaluating autonomic markers alongside structural and metabolic risk factors may clarify their prognostic value.

The relationship between psychological stress, sleep disturbance, and cardiovascular outcomes also requires further study. Although PTSD and chronic stress are prevalent among first responders,^10-14^ direct associations with objective cardiovascular endpoints in firefighters remain incompletely defined. Evaluation of targeted interventions addressing psychological stress and sleep quality may help determine their role in cardiovascular risk modification.

Finally, implementation research is needed to define practical strategies for integrating guideline-directed cardiovascular screening and management within fire service settings. Evaluation of structured programs to improve blood pressure control, lipid management, and adherence to preventive therapies may inform approaches to occupational cardiovascular risk reduction.

Addressing these areas will require collaboration across cardiovascular medicine, occupational health, and behavioral science disciplines.

## 9. Conclusion

Cardiovascular disease remains the leading cause of line-of-duty mortality among firefighters. Available data suggest that sudden cardiac death in this population occurs predominantly in individuals with underlying cardiovascular disease and is frequently triggered during periods of high physiologic demand. Coronary atherosclerosis and hypertension provide the structural basis for disease, while acute exertion and physiologic stress contribute to event precipitation.

These observations are consistent with established mechanisms of ischemic and arrhythmic death rather than a distinct occupational pathology, and support the need for earlier and more aggressive cardiovascular risk stratification in firefighters, potentially incorporating imaging-based approaches such as coronary artery calcium scoring in selected individuals. Accordingly, emphasis on early identification of modifiable risk factors, implementation of guideline-directed therapy, and attention to occupational contributors such as stress and sleep disruption represent a rational approach to cardiovascular risk reduction in this population.
